# Thrombomodulin is a stronger indicator of combined oral contraceptives-induced activated protein C pathway resistance in the thrombin generation test than activated protein C

**DOI:** 10.3389/fcvm.2024.1490601

**Published:** 2024-11-29

**Authors:** Marisa Ninivaggi, Lily Sokolova, Demy Donkervoort, Bas de Laat, Romy de Laat-Kremers

**Affiliations:** ^1^ Department of Functional Coagulation, Synapse Research Institute, Maastricht, Netherlands; ^2^ Department of Data Analysis and Artificial Intelligence, Synapse Research Institute, Maastricht, Netherlands

**Keywords:** thrombin, activated protein c, thrombomodulin, coagulation, combined oral contraceptives

## Abstract

**Background:**

The mechanism by which combined oral contraceptives (COCs) lead to hypercoagulation is not fully understood, although activated protein C (APC) pathway resistance has been implicated. APC and thrombomodulin (TM) tend to be considered as interchangeable reagents, even though their biological action in coagulation is different. However, it remains unclear which reagent is better suited for the detection of APC pathway resistance. We compared the effectiveness of TM and APC in TG to detect COC-induced APC pathway resistance using thrombin generation (TG).

**Methods:**

TG was measured on ST Genesia in 48 healthy women, of whom 24 used COCs. TG was triggered with STG-ThromboScreen (with and without TM), spiked with a low and high concentration of TM or APC (2 or 15 nM TM, or 1.5 or 5.5 nM APC), aimed to achieve 50% and 90% ETP inhibition, respectively.

**Results:**

TG was higher in women using COCs. TM and APC inhibit TG in all women, although their inhibitory effect is more pronounced in women without COC compared to women with COC. The addition of 2 nM TM causes an ETP reduction of 40% (1,289 vs. 768 nM•min) in women without COC and an ETP reduction of 24% (1,704 vs. 1,287 nM•min) in women with COC. The addition of 1.5 nM APC causes an ETP reduction of 41% (1,289 vs. 759 nM•min) in women without COC and an ETP reduction of 23% (1,704 vs. 1,316 nM•min) in women with COC. The difference in effect between women with and without COC is largest when 15 nM TM, aimed at 90% ETP inhibition, is used. 15 nM TM leads to the smallest overlap in ETP inhibition between women with and without COC (27% overlap), compared to 2 nM TM (41% overlap), and 1.5 nM APC (38% overlap) and 5.5 nM APC (41% overlap).

**Conclusion:**

Although TM and APC are often used interchangeably to assess the sensitivity of the APC system in TG, our findings suggest that TM is a better discriminator to detect COC-use induced APC pathway resistance. In addition, we found that the ETP is a better TG test readout for APC pathway resistance testing than the peak height.

## Introduction

Combined oral contraceptive (COC) use is associated with an increased risk of venous thromboembolism (VTE) (1). COCs contain both estrogen and progestin, in contrast to progestin only contraceptives (2). The type of progestin in the COC modulates the COC-associated risk of thrombosis. Women using a third generation COC containing desogestrel have a higher risk of thrombosis compared to women using a second generation COC containing levonorgestrel (3). This is reflected in a more pronounced attenuation of the APC pathway in women using a COC containing desogestrel (4).

The mechanism by which COCs lead to hypercoagulation is not yet fully understood (5). Some studies report a decrease in antithrombin and protein S, with a concomitant increase in prothrombin and factors VII and VIII (6, 7); while others report no increase in plasma levels of prothrombin or thrombin-antithrombin complexes (8). Another well-studied topic in relation to the pro-coagulant nature of COCs is the activated protein C (APC) pathway (9–13). Thrombomodulin (TM) is an endothelial cell-derived surface protein, which binds to and enables thrombin to activate protein C. APC in turn associates with protein S and the complex downregulates factors Va and VIIIa, thereby limiting further amplifications of the coagulation cascade and serving as a protective mechanism from pathological hypercoagulation (14, 15). When this pathway is desensitized, for example due to COC use, a resistance to the TM—APC pathway occurs (16).

Over the last decades, the APC pathway has become a research subject of interest (7, 10, 17, 18). Especially as the prothrombotic effect of widely used combined oral contraceptives are known to be at least in part provoked by inhibition of the APC pathway (10), several assays have been developed to quantify the sensitivity of the APC pathway in an individual (19–23). In clinical laboratories, resistance to APC and/or TM is commonly studied using coagulation time tests or the thrombin generation (TG) assay, both performed in the absence and presence of TM and/or APC (19, 24). The difference in test-readout between the two conditions are used to calculate the APC sensitivity ratio, which is an indicator of APC resistance (24). TM and APC are currently used interchangeably to determine APC resistance, although difference in effect between the two proteins in the TG assay is not completely understood. Both TM and APC have been used to investigate the APC pathway in the calibrated automated thrombinography (CAT) assay (6, 20). The ST Genesia platform uses the Thromboscreen kit containing TG-trigger reagent with and without TM, which is commercially available (25, 26). Typically, both TM and APC reagents are aimed at achieving a reduction of TG by 50%, either based on the ETP or peak height, although it has recently been suggested for APC that aiming for 90% inhibition of the ETP could be of interest as well (27).

Recently the question arose whether APC resistance can better be studied using TM, APC or both reagents, and which concentration should be used (24). Specifically for women using COCs, it would be interesting to know which reagent is a better discriminator to detect an APC-pathway related pro-coagulant phenotype in the TG assay. Therefore, we investigated whether the addition of TM or APC in the TG assay would result in different sensitivity for detecting APC resistance in COC users.

## Materials and methods

### Study population and sample collection

Healthy subjects were enrolled in the study after giving written informed consent and in accordance with the Declaration of Helsinki. Exclusion criteria were male sex, age above 65 years, age below 18 years, pregnancy, known coagulation defects, and the use of anticoagulant medication. The study population consisted of 48 healthy women, of whom 24 used COC. Seventy percent of women used COCs containing ethinylestradiol and levonorgestrel, whereas 30 percent used COC containing ethinylestradiol and desogestrel. Subjects were age-matched, with an average age of 25.3 ± 5.8 years in women without COC, and an average age of 25.4 ± 6.7 years in women using COC.

For the optimization of TM and APC concentration prior to this study, healthy donor samples were collected. For this population, exclusion criteria were age above 65 years, age below 18 years, pregnancy, COC use, known coagulation defects, and the use of anticoagulant medication.

Blood was collected in vacuum blood drawing tubes (Greiner Bio-One) containing 3.2% citrate in a 9:1 ratio. Platelet poor plasma (PPP) was prepared by centrifuging twice at 2,821·g for 10 min, in accordance with the ISTH-SSC guidelines (28). PPP was stored at −80°C until for a maximum of 2 years (28) and were thawed by placing the samples in a warm water bath (37°C) prior to the start measurement.

### Thrombin generation measurements

Thrombin generation was measured on the fully automated ST Genesia using the STG-ThromboScreen reagent kit according to the manufacturer's recommendations (Diagnostica Stago, France). The STG-ThromboScreen reagent kit contains two types of TG-trigger reagents: one reagent contains tissue factor and phospholipids, whereas the other reagent contains thrombomodulin (TM) in addition to tissue factor and phospholipids. As in the semi-automated CAT assay, the first reagent is used to measure “normal” thrombin generation, whereas the second reagent is used to assess the sensitivity of the anticoagulant activated protein C pathway (20). The latter condition is typically analyzed by calculating the percentage inhibition of either the ETP or the peak height by the inhibitory actions of TM. In addition to the commercially provided reagents, in this study, we spiked the STG-ThromboScreen reagent (without TM in the original reagent) with 2 or 15 nM (f.c.) in-house recombinant TM (Synapse Research Institute, the Netherlands), or 1.5 and 5.5 nM (f.c.) activated protein C (Synapse Research Institute, the Netherlands). TM was produced as a human recombinant protein, with the same sequence of the TM of Asahi Kasei. APC was produced in-house as previously described by Regnault et al. (29). The concentrations of TM and APC were chosen based on a respective inhibition of TG of 50% and 90% for both inhibitors. The inhibitory potential of both APC and TM were determined in pooled normal plasma upon production of the batch of each reagent. Prior to this study, the inhibitory potential of TM and APC was confirmed in 8 healthy donors (4 men, 4 women not using OC) achieved in a reference group of healthy individuals, including men and women. The intra- and inter-assay coefficients of variation were 1.6% and 2.2%, respectively for lag time, 4.3% and 4.9% for peak height, 1.3% and 2.9% or time-to-peak, 6.7% and 2.7% for ETP, and 3.2% and 8.6% for velocity index. Due to the number of tests performed, the TG measurements were performed on 6 days, using the same ST Genesia device. Individuals with and without OCs were equally distributed over the measurement days to avoid day-to-day bias in the samples. The variation between day-to-day controls was 1.7%.

### Analysis of TG inhibition by APC and TM

Percentage inhibition by TM or APC was calculated as stated in Equations 1–4, respectively for peak height (PH), and ETP.

(1) $$\frac{\Delta \mathrm{Peak}\;\mathrm{height}}{\mathrm{Peak}\;\mathrm{heigh}t_{\mathrm{AP}C^{-}}}\cdot 100\%\;\mathrm{with}\;\Delta \mathrm{Peak}\;\mathrm{height}=\mathrm{Peak}\;\mathrm{heigh}t_{\mathrm{AP}C^{-}}-\mathrm{Peak}\;\mathrm{heigh}t_{\mathrm{AP}C^{+}}$$

(2) $$\frac{\Delta \mathrm{Peak}\;\mathrm{height}}{\mathrm{Peak}\;\mathrm{heigh}t_{TM^{-}}}\cdot 100\%\;\mathrm{with}\;\Delta \mathrm{Peak}\;\mathrm{height}=\mathrm{Peak}\;\mathrm{heigh}t_{TM^{-}}-\mathrm{Peak}\;\mathrm{heigh}t_{TM^{+}}$$

(3) $$\frac{\Delta \mathrm{ETP}}{\mathrm{ET}P_{\mathrm{AP}C^{-}}}\cdot 100\%\;\mathrm{with}\;\Delta \mathrm{ETP}=\mathrm{ET}P_{\mathrm{AP}C^{-}}-\mathrm{ET}P_{\mathrm{AP}C^{+}}$$

(4) $$\frac{\Delta \mathrm{ETP}}{\mathrm{ET}P_{TM^{-}}}\cdot 100\%\;\mathrm{with}\;\Delta \mathrm{ETP}=\mathrm{ET}P_{TM^{-}}-\mathrm{ET}P_{TM^{+}}$$

### Statistics

Statistical analysis was performed using GraphPad Prism Version 10 (GraphPad Prism, USA). Distribution normality was tested using the Kolmogorov-Smirnov test. Differences between women with and without COC were analyzed by a Student *t*-test or a Mann-Whitney test, depending on the distribution on the data. Differences between the effect of APC and TM addition on TG parameters was analyzed by ANOVA or Friedman analysis with *post hoc* correction, depending on data distribution. Glass' delta was used to determine the effect size of COC use on the inhibition of ETP and peak height by TM and APC.

## Results

**Figure 1:**
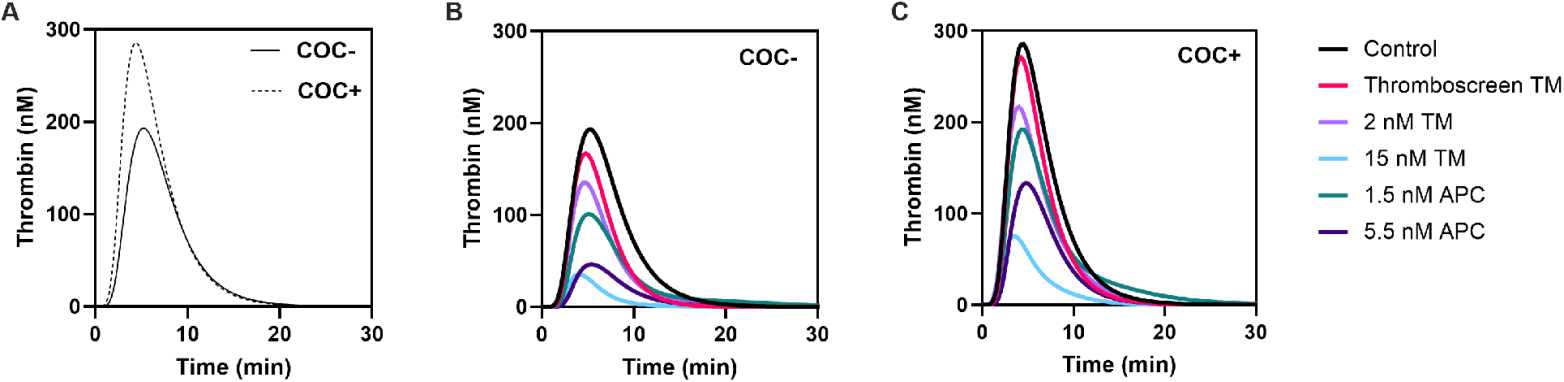
Average TG curves in women with and without combined oral contraceptives and the effect of added TM and APC. **(A)** TG curves in women without COC (continuous line) and women with COC (dashed line). **(B-C)** Effect of Thromboscreen TM, 2 nM and 15 nM in-house TM, and 1.5 nM and 5.5 nM in-house APC in women without COC **(B)** and women with COC **(C).**

**Figure 2:**
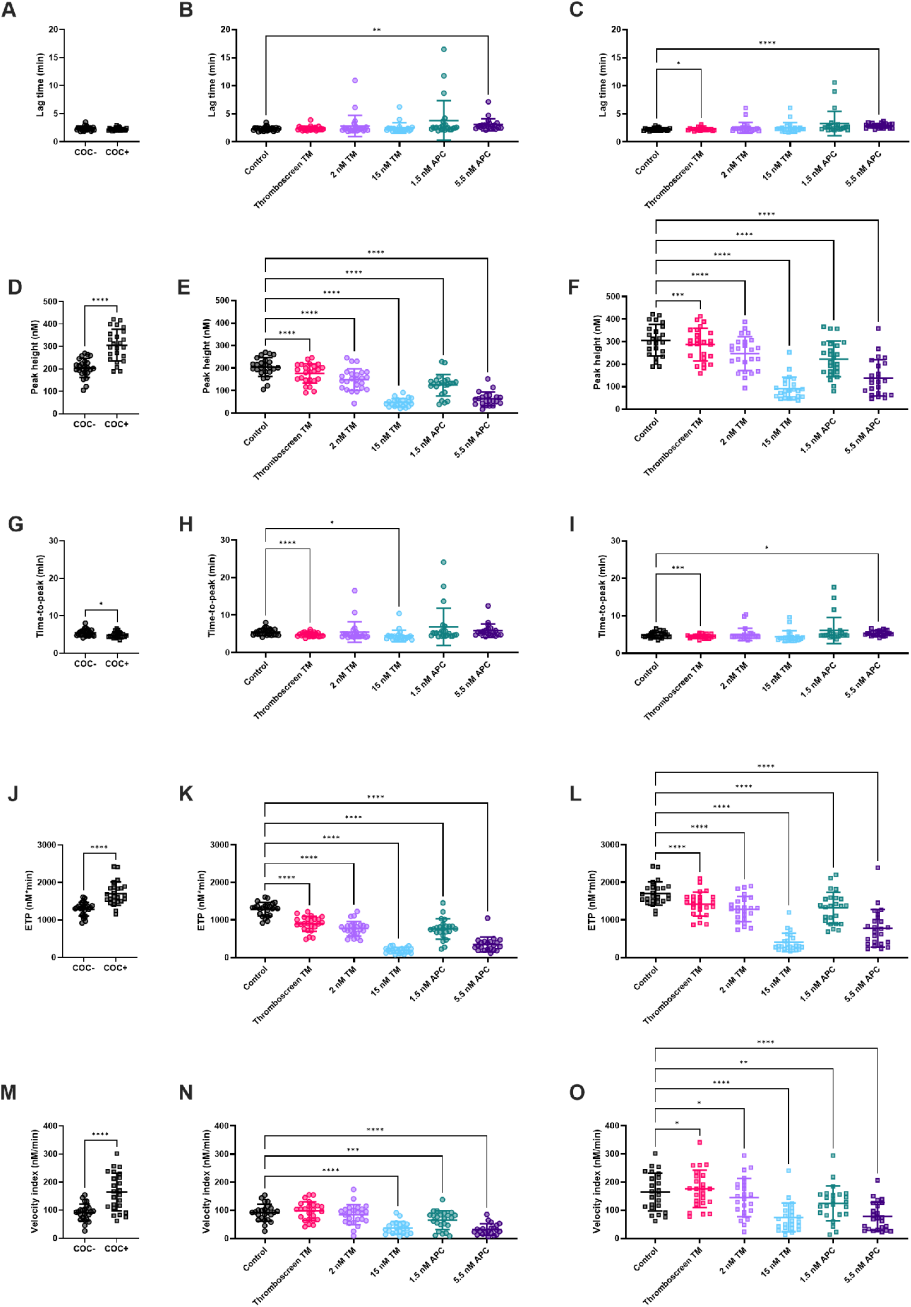
The effect of TM and APC on TG parameters in women with and without combined oral contraceptives. **(A)** TG lag time was comparable in women with and without COC. **(B,C)** The effect of TM and APC on lag time in women without COC **(B)**, and with COC **(C)**. **(D)** Peak height was significantly higher with COC compared to women without COC. (E-F) The effect of TM and APC on peak height in women without **(E)** and with COC **(F)**. **(G)** Time-to-peak was significantly shorter in women with COC compared to women without COC. **(H,I)** The effect of TM and APC on time-to-peak in women without COC **(H)** and women with COC **(I)**. **(J)** ETP was significantly higher in women with COC compared to women without COC. **(K,L)** The effect of TM and APC on ETP in women without COC **(K)** and women with COC **(L)**. **(M)** Velocity index was significantly higher in women with COC compared to women without COC. **(N,O)** The effect of TM and APC on velocity index in women without COC **(N)** and women with COC **(O)**. Data are shown as individual values with mean and standard deviation. *p < 0.05, **p < 0.01, ***p < 0.001, ****p < 0.0001. APC, activated protein C; ETP, endogenous thrombin potential; TM, thrombomodulin.

TG was measured in 24 women without COCs (COC-) and 24 women with COC (COC+) on ST Genesia using the Thromboscreen reagent (Figures 1, 2). The TG curve in women with COC as above the TG curve in women without COC (Figure 1A). TG curves were further quantified by the lag time, peak height, time-to-peak, ETP and velocity index (Figure 2). The use of COC resulted in a significantly higher peak height (+49%, *p* < 0.001, Figure 2D), ETP (+32%, *p* < 0.001, Figure 2J) and velocity index (+78%, *p* < 0.001, Figure 2M) and a shorter time-to-peak (−11%, *p* = 0.019, Figure 2G). The commercial TM reagent caused a statistically significant inhibition of the TG curve, both in women with and without COC, although the inhibitory effect was more pronounced in women without COC (Figures 1B,C). In addition, 2 nM and 15 nM TM were added to the Thromboscreen reagent, achieving 50% and 90% inhibition of the ETP, as previously determined in a healthy reference population. Similarly, 1.5 and 5.5 nM APC, were added to the Thromboscreen reagent, achieving 50% and 90% inhibition of the ETP. Both the addition of in-house preparations of TM and APC caused a significant reduction of the TG curve in women with and without COCs (Figures 1B,C).

### Effect of Thrombomodulin and activated protein C on TG

Figure 2 shows the quantification of TG in the absence and presence of TM or APC. Thromboscreen containing kit-derived TM caused a significant reduction of TG peak height (205 nM vs. 176 nM, *p* < 0.0001) and ETP (1,289 nM•min vs. 896 nM •min, *p* < 0.0001) in women without COC compared to TG measured using Thromboscreen without added TM. In women, with COC, thromboscreen containing kit-derived TM caused a significant reduction of TG peak height 306 nM vs. 287 nM, *p* < 0.0001) and ETP (1,704 nM•min vs. 1,423 nM•min, *p* < 0.0001) as well. The time-to-peak was significantly shortened by kit-derived TM in women with and without COC (4.9 min vs. 4.5 min, 5.4 min vs. and 4.8 min, respectively). In addition, in women with COC, lag time was slightly prolonged (2.2 min vs. 2.3 min, *p* < 0.01) and the velocity index was increased (164 nM/min vs. 175 nM/min, *p* = 0.03).

We further investigated the effect of higher concentrations of (in-house preparations of) TM on TG parameters in women with and without COC. In women without COC, 2 and 15 nM TM significantly reduced ETP (1,289 nM•min vs. 768 nM•min and 188 nM•min, respectively, both *p* < 0.0001)) and peak height (205 nM vs. 150 nM and 47 nM, respectively, both *p* < 0.0001). In women with COC, 2 and 15 nM TM significantly reduced ETP (1,704 nM•min vs. 1,287 nM•min and 401 nM•min, respectively, both *p* < 0.0001)) and peak height (306 nM vs. 247 M and 92 nM•min, respectively, both *p* < 0.0001). In addition, the higher TM concentration of 15 nM significantly decreased the velocity index, regardless of COC use.

APC significantly inhibited TG peak height, ETP and velocity index, regardless of whether women used COC or not. In more detail, 1.5 nM APC caused a reduction of peak height (205 nM vs. 124 nM, *p* < 0.0001), ETP (1,289 nM•min vs. 759 nM•min, *p* < 0.0001) and velocity index (92 nM/min vs. 84 nM/min, *p* = 0.0003) in women without COC. In women with COC, 1.5 nM APC caused a reduction of peak height (306 nM vs. 223 nM, *p* < 0.0001), ETP (1,704 nM•min vs. 1,316 nM•min, *p* < 0.0001) and velocity index (164 nM/min vs. 145 nM/min, *p* = 0.0047). A higher concentration of APC (5.5 nM), cause a significantly larger inhibition of peak height (205 nM vs. 63 nM, *p* < 0.0001), ETP (1,289 nM•min vs. 347 nM•min, *p* < 0.0001) and velocity index (92 nM/min vs. 37 nM/min, *p* < 0.0001) in women without COC, and women with COC (peak height: 306 nM vs. 139 nM, *p* < 0.0001; ETP: 1,704 nM•min vs. 775 nM•min, *p* < 0.0001; velocity index: 164 nM/min vs. 74 nM/min, *p* < 0.0001). Furthermore, 5.5 nM APC significantly prolonged the lag time in both groups by +55% on average, whereas the time-to-peak was only prolonged in women using COC (+10%, *p* = 0.01).

### Inhibition of ETP and peak height by TM and APC

**Figure 3:**
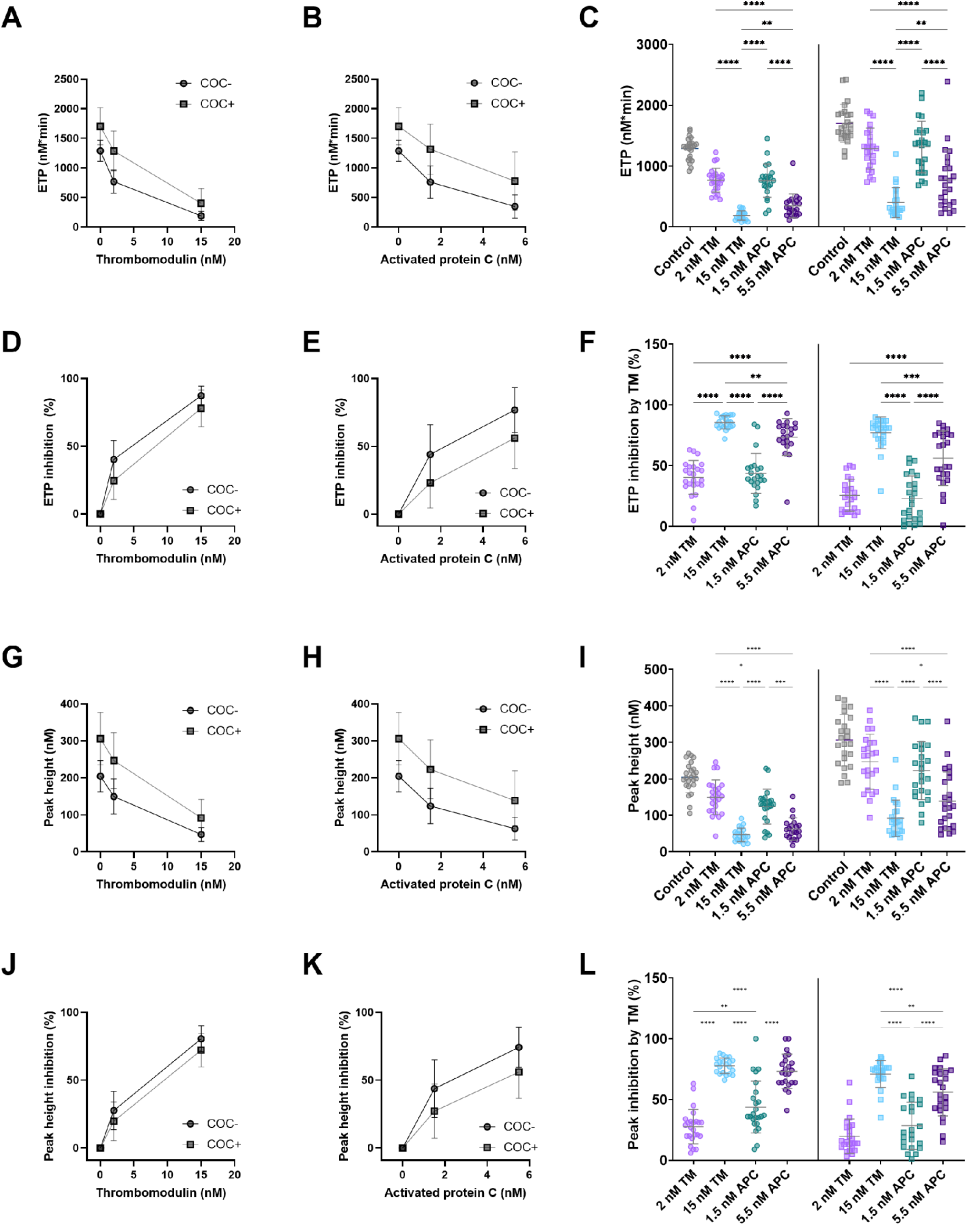
The dose-dependent effect of in-house TM and APC on ETP and peak height. **(A)** The absolute inhibitory effect of 2 and 15 nM TM on ETP in women with and without COC. **(B)** The absolute inhibitory effect of 1.5 and 5.5 nM APC on ETP in women with and without COC. **(C)** The absolute inhibition of the ETP by TM and APC. **(D)** The relative inhibitory effect of 2 and 15 nM TM on ETP in women with and without COC. **(E)** The relative inhibitory effect of 1.5 and 5.5 nM APC on ETP in women with and without COC. **(F)** The percentage inhibition of the ETP by TM and APC. **(G)** The absolute inhibitory effect of 2 and 15 nM TM on peak height in women with and without COC. **(H)** The absolute inhibitory effect of 1.5 and 5.5 nM APC on peak height in women with and without COC. **(I)** The absolute inhibition of the peak height by TM and APC. **(J)** The relative inhibitory effect of 2 and 15 nM TM on peak height in women with and without COC. **(K)** The relative inhibitory effect of 1.5 and 5.5 nM APC on peak height in women with and without COC. **(L)** The percentage inhibition of the peak height by TM and APC. Data are shown as (individual values with) mean and standard deviation. *p < 0.05, **p < 0.001, ***p < 0.001, ***p < 0.0001. APC, activated protein C; ETP, endogenous thrombin potential; COC, combined oral contraceptives; TM, thrombomodulin.

The effect of TM and APC was further quantified as the percentage inhibition of the TG peak height and ETP (Figure 3). Both TM and APC inhibit ETP (Figures 3A–C) and peak height (Figures 3D–F) in a dose-dependent manner. The addition of 2 nM TM caused an ETP inhibition of 40% and 26%, respectively in women without and with COC (Figure 3C). A higher TM concentration (15 nM) resulted in an ETP reduction of 86% and 77% in women without and with COC. Similarly, 1.5 nM APC and 5.5 nM APC respectively reduced the ETP by 44% and 73% in women without COC, and by 23% and 56% in women with COC (Figure 3F). In addition, we quantified the inhibitory effect of TM and APC on the peak height. The lower concentration of TM (2 nM) caused a peak height inhibition of 28% and 20% in women without and with COC, respectively (Figure 3C), whereas 15 nM TM resulted in a peak height reduction of 78% and 71% in women without and with COC. Moreover, 1.5 nM APC and 5.5 nM APC respectively reduced peak height by 44% and 73% in women without COC, and by 28% and 56% in women with COC.

### Differentiation between subjects with and without COC by TM and APC

**Figure 4:**
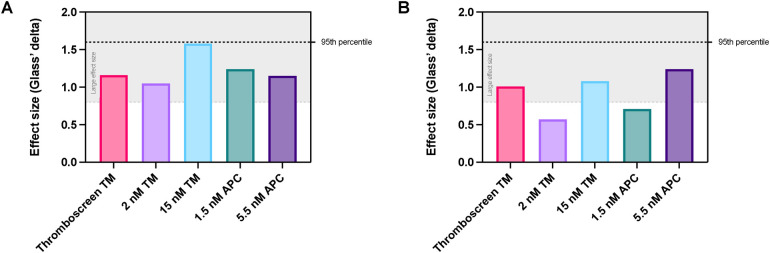
Comparison of the effect size of TM and APC addition for the discrimination between women with and without COC. **(A)** Effect size of the inhibition of ETP in women with vs. without COC when using TM or APC. **(B)** Effect size of the inhibition of peak height in women with vs. without COC when using TM or APC. Data are shown as the Glass's delta statistic for each reagent when comparing COC- and COC + women. The grey area in the graph indicates Glass's delta statistic values that are considered to be a large effect. *APC, activated protein C; ETP, endogenous thrombin potential; TM, thrombomodulin*.

We further analyzed the differentiation potential of each reagent type and concentration to distinguish between women with and without COC (Figure 4). We compared the effect size of ETP inhibition (Figure 4A) and peak height inhibition (Figure 4B) of each type and concentration of reagent in women with and without COC. When using ETP as a test read-out, all TM- and APC-containing reagents cause a large difference in effect between women without and with COC (Figure 4A), ranging from 27% to 41% overlap between the COC + and COC- groups. Interestingly, the difference in effect is lower if the peak height is used as a read-out variable, ranging from 38% to 77% overlap between the groups (Figure 4B), indicating that the ETP is a better read-out for APC sensitivity testing than peak height. Interestingly, the inhibition of the ETP by 15 nM TM (i.e., the equivalent that induces 90% ETP inhibition in a general healthy population) caused the best discrimination between the groups of women with and without COC.

## Discussion

The assessment of APC pathway resistance is of clinical interest as widely used combined oral contraceptives are known to desensitize the APC pathway (30). Several methods, similar to some extent, have been developed to quantify the sensitivity of the APC pathway, in a so called APC resistance assay. APC pathway resistance can be quantified by the addition of activated protein C directly, circumventing the activation of prothrombin C by thrombomodulin-bound thrombin. Alternatively, the function of the activated protein C pathway can be triggered by the addition of thrombomodulin, which induces thrombin to activate the protein C present in the plasma sample to activate the APC-pathway (7, 10, 17, 18).

In the current study, we investigated the diagnostic potential of the addition of either APC or TM in the thrombin generation test executed on the ST Genesia. Thrombin generation was measured in the presence and absence of APC and TM in a cohort of 48 women, of whom 24 used COC. The potential of TM- and APC-induced inhibition of TG as a diagnostic tool for the detection of APC pathway resistance was compared.

We found that COC use induces a hypercoagulant TG profile, even in the absence of a trigger for the APC pathway, which is consistent with previous studies (6, 10). The inhibition of ETP and peak height by TM or APC was lower in women using COC compared to women without COC, which is well documented in literature (25, 26). Most studies on the association of COC use and TG primarily focus on APC pathway resistance in general (25, 26, 31). APC and TM are often used interchangeably to some extent to test the sensitivity of the APC system in the thrombin generation assay (20, 31), the diagnostic superiority of either APC or TM has not been studied (24). Although the biological effects of APC and TM on TG are interconnected mechanistically (18), it has not yet been addressed to which extent APC and TM affect TG results individually.

In this study, we found that the ETP is a better TG test readout for APC pathway resistance testing than the peak height. Furthermore, our results suggest that the inhibition of TG by the high TM concentration, that induces 90% ETP inhibition in a general healthy population, leads to the best discrimination between the groups of women with and without COC. Douxfils et al. indeed previously hypothesized that a higher concentration of APC aimed at 90% inhibition of the ETP might be superior in the detection of COC-associated APC resistance (22, 24). Unfortunately, they did not consider TM in their assay. We now show that the use of a higher TM concentration, aimed at 90% ETP inhibition in healthy subjects, could be an even better choice of reagent than 90% APC, as this results in a better discrimination between women with and without COC. A higher concentration of TM had not only show the strongest inhibitory effect, it also caused a more reproducible inhibition in terms of a smaller standard deviation for the effect of TM. Further studies are required to confirm our current findings, and address the diagnostic potential of both APC and TM in thrombin generation for the detection of APC pathway resistance.

The interference of TM and APC in the coagulation system occurs at different levels. The addition of APC is the mere addition of an anticoagulant that directly inhibits Factor Va and VIIIa, and thereby reduces the TG curve (17). In contrast, the addition of TM elicits the activation of a chain of reactions by binding to the thrombin that is formed during the TG experiment (20). The binding of thrombin to thrombomodulin evokes its anticoagulant action, by activating protein C present in the plasma sample, which in turn inactivates Factors Va and VIIIa (20). Therefore, TM addition to a TG experiment, does not only examine the effect of FVa and FVIIIa inhibition, but also the processes underlying the APC pathway, including the potential of a plasma sample to generate thrombin—which binds to thrombomodulin in order to form the APC generating complex—and the amount of protein C that is available.

As a result, the application of the assay in part determines which trigger would be more suitable in a certain assay, i.e., to measure the resistance to APC (APC resistance) or the resistance to the activation of the APC pathway by TM (APC pathway resistance). The addition of TM would result in a broader testing of the APC pathway, although it would also be expected to have more interference from other coagulation processes upstream of the formation of thrombin, and he level of protein C. Additionally, Zermatten et al. previously suggested that the addition of TM to the TG test could be a promising tool to identify women taking combined oral contraceptives at high risk for venous thromboembolism, for example due to an unknown FV_Leiden_ mutation (32).

This pilot study on the use of TM and APC for APC pathway sensitivity testing in women on COCs has several limitations. Even though the size of the study cohort used in this pilot study is small, and the results should be confirmed in a larger cohort, the statistical power was sufficient to achieve meaningful results. Moreover, in this study, we investigated the effect of different concentrations of TM and APC, to shed light on their similarities and differences with respect to the design of diagnostic assays. Although the currently available data is not sufficient to make a final decision on the design of the perfect diagnostic assay for the evaluation of COC-associated thrombosis risk on an individual level, these data contribute to its design and development. In addition, we were unable to measure plasma protein C levels due to insufficient plasma volumes.

In conclusion, our study shows that although TM and APC are often used interchangeably to assess the sensitivity of the APC system in TG, TM is a better discriminator to detect COC-use induced APC resistance in our study population.

As the effect of hormonal treatment, including COC use, on the mechanism of the APC pathway is poorly understood, it is difficult to determine the reason for the higher sensitivity of the system to TM over APC at higher concentration. An explanation for this finding could be the different mode of action of TM and APC in the coagulation cascade. We propose that the use of TM allows to assess the whole APC pathway, including its activation by the thrombin-TM complex, the protein C level in the plasma and the subsequent response in inhibition of FV_a_ and FVIII_a_ by the generated APC.

## Data Availability

The original contributions presented in the study are included in the article/Supplementary Material, further inquiries can be directed to the corresponding author.
